# DB-02, a C-6-Cyclohexylmethyl Substituted Pyrimidinone HIV-1 Reverse Transcriptase Inhibitor with Nanomolar Activity, Displays an Improved Sensitivity against K103N or Y181C Than *S*-DABOs

**DOI:** 10.1371/journal.pone.0081489

**Published:** 2013-11-25

**Authors:** Xing-Jie Zhang, Li-He Lu, Rui-Rui Wang, Yue-Ping Wang, Rong-Hua Luo, Christopher Cong Lai, Liu-Meng Yang, Yan-Ping He, Yong-Tang Zheng

**Affiliations:** 1 Key Laboratory of Animal Models and Human Disease Mechanisms of the Chinese Academy of Sciences & Yunnan Province, Kunming Institute of Zoology, Chinese Academy of Sciences, Kunming, Yunnan 650223, P. R. China; 2 University of Chinese Academy of Sciences, Beijing, P. R. China; 3 Key Laboratory of Medicinal Chemistry for Natural Resource Ministry of Education, School of Chemical Science and Technology, Yunnan University, Kunming, Yunnan, P. R. China; 4 Chemical Biology Laboratory, Center for Cancer Research, National Cancer Institute, National Institutes of Health, Frederick, Maryland, United States of America; Institut Pasteur of Shanghai,Chinese Academy of Sciences, China

## Abstract

6-(cyclohexylmethyl)-5-ethyl-2-((2-oxo-2-phenylethyl)thio)pyrimidin-4(3H)-one (DB-02) is a member of the newly reported synthetic anti-HIV-1 compounds dihydro-aryl/alkylsulfanyl-cyclohexylmethyl-oxopyrimidines, *S*-DACOs. *In vitro* anti-HIV-1 activity and resistance profile studies have suggested that DB-02 has very low cytotoxicity (CC_50_>1mM) to cell lines and peripheral blood mononuclear cells (PBMCs). It displays potent anti-HIV-1 activity against laboratory adapted strains and primary isolated strains including different subtypes and tropism strains (EC_50_s range from 2.40 to 41.8 nM). Studies on site-directed mutagenesis, genotypic resistance profiles revealed that V106A was the major resistance contributor for the compound. Molecular docking analysis showed that DB-02 located in the hydrophobic pocket with interactions of Lys101, Val106, Leu234, His235. DB-02 also showed non-antagonistic effects to four approved antiretroviral drugs. All studies indicated that DB-02 would be a potential NNRTI with low cytotoxicity and improved activity.

## Introduction

Acquired immunodeficiency syndrome (AIDS) was first reported in 1981 and currently there are 34 million people living with the infection [1]. Highly active antiretroviral therapy (HAART) is a popular method used to control AIDS and to reduce the mortality of the patients. Nonnucleoside reverse transcriptase inhibitors (NNRTIs) are the major components of HAART in clinical therapy. NNRTIs are hydrophobic molecules with diverse chemical structures that are generally highly specific for HIV-1 [2]. Compare with nucleoside reverse transcriptase inhibitors (NRTIs), NNRTIs exhibit higher selectivity and efficacy to HIV-1 [3,4]. However, the rapid emergence of mutations, such as K103N and Y181C mutations, has decreased the efficiency of the treatment and often leads to failure of the therapy [5]. This adverse effect reduced the clinical usage of first generation NNRTIs. More effective second-generation NNRTIs, etravirine and rilpivirine, were developed to overcome this difficulty. However, they are not available in high prevalence AIDS countries, such as China, due to their high costs. Therefore, it is necessary to develop new NNRTIs with lower costs and wider availability.

**Figure 1:**
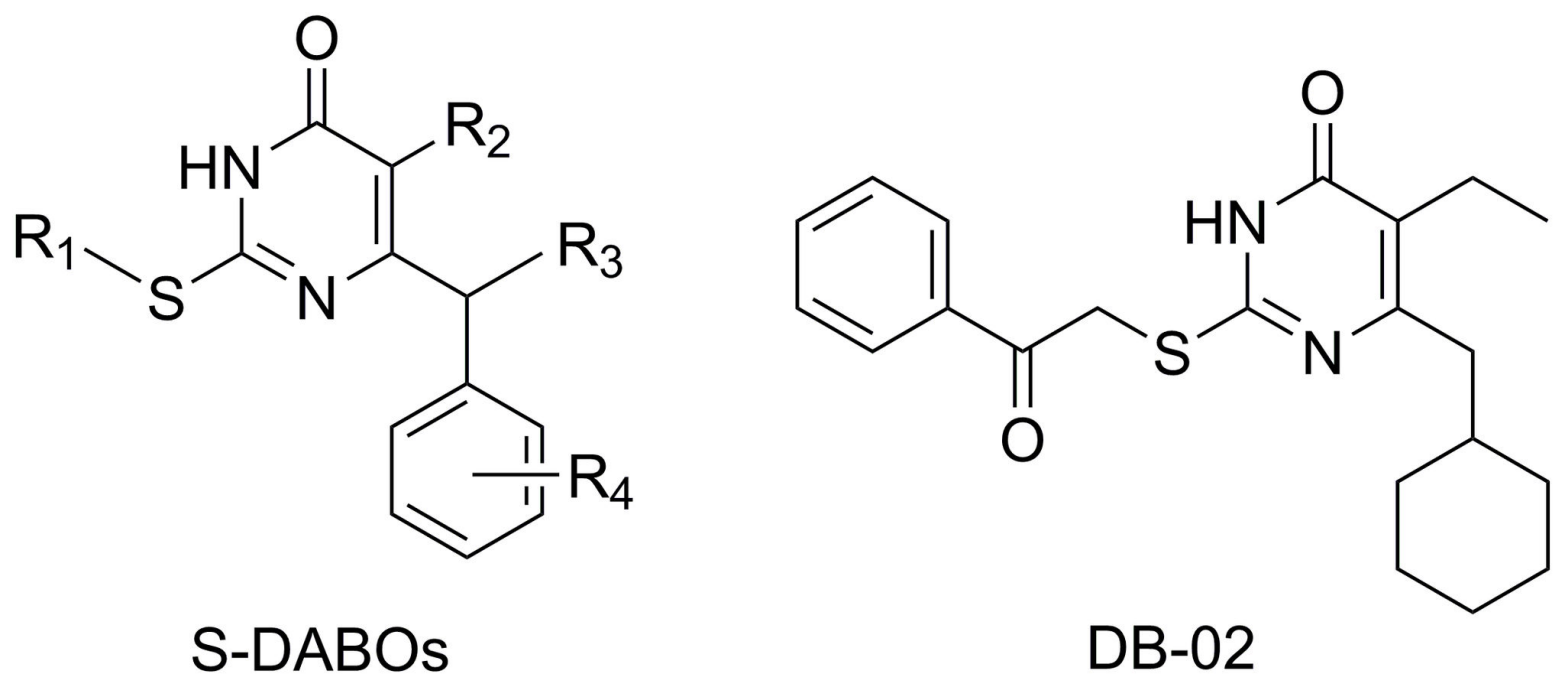
Structures of *S*-DABO and DB-02.

Dihydroalkylthiobenzyloxopyrimidines (*S*-DABOs) is a novel reservoir of NNRTIs with unique antiviral potency, high specificity, and low toxicity [6-9]. However, the antiviral activity of *S*-DABOs is easily prone to be decreased by some single-site mutations, such as K103N or Y181C. As an improvement of *S*-DABOs, our recent work obtained a series of novel dihydro-aryl/alkylsulfanyl-cyclohexylmethyl-oxopyrimidines (*S*-DACOs) with improved antiviral activities against HIV-1. Among them, 6-(cyclohexylmethyl)-5-ethyl-2-((2-oxo-2-phenylethyl)thio) pyrimidin-4(3H)-one (DB-02, see Figure 1) exhibited high potent activity [10]. 

In this continued DB-02 evaluation study, we put more effort on the *in vitro* cytotoxicity and antiviral activity of DB-02 on different cell lines, including different subtype strains, clinical strains, and resistant strains. We also tested the reverse transcriptase (RT) activity, site-directed mutation (SDM) virus susceptibility, phenotypic and genotypic resistance of DB-02 treated cells. Drug combination activity and molecular docking results of DB-02 are also reported.

## Materials and Methods

### Ethics statement

Ethical approval for the study and the informed consent process were approved by the Ethics Committee of Kunming Institute of Zoology, Chinese Academy of Sciences (Approval Number: SWYX-2009012, 2009013). Written informed consent was obtained from all involved participants prior to the study. The study was conducted in accordance with basic principles of the Helsinki declaration and the relevant international rules.

### Compounds and reagents

DB-02 was synthesized as described previously (Figure 1) [10]. Dimethyl sulfoxide (DMSO), azidothymidine (AZT), 3-(4, 5-Dimethyl-2-thiazolyl)-2, 5-diphenyl-2H-tetrazolium bromide (MTT), sodium dodecyl sulfate (SDS), N, N-dimethylformamide (DMF), phytohemagglutinin (PHA) and interleukin-2 (IL-2), were purchased from Sigma-Aldrich company (MO, USA). Raltegravir (RAL) was obtained from Selleck Chemicals (Houston, TX, USA). Nevirapine (NVP), efavirenz (EFV) was purchased from US Pharmacopeia (Rockville, MD, USA). Etravirine (ETR) was obtained from Santa Cruz Biotechnology (CA, USA).

### Cells and viruses

C8166, MT-4 and H9 cells were kindly provided by the AIDS Reagent Project, the UK Medical Research Council (MRC). Laboratory adapted strains, including HIV-1_IIIB_, and HIV-1_MN_, and HIV-1 reverse transcriptase (RT) resistant strains, including HIV-1_A17_ and HIV-1_L74V_, were obtained from the NIH AIDS Research and Reference Reagent Program (USA). Clinical isolated HIV strains, including HIV-1_KM018_, HIV-1_TC-2_ and HIV-1_WAN_ were isolated from local AIDS patients in Yunnan, China before antiviral drug treatment (Ethical Approval Number: SWYX-2009012). PBMCs were isolated by Ficoll-Hypaque method from whole blood collected from healthy donor (Ethical Approval Number: SWYX-2009013).

### Cytotoxicity assays

Cytotoxicity was assayed by MTT colorimetric reduction as previously described with some modifications [11]. Briefly, 100 μl 4×10^4^ C8166 or MT-4 cells were added in a 96-well plate, then a series of concentrations of DB-02 were added in each well (100 μl per well). After 3 days of incubation at 37°C, 5% CO_2_, the cell viability was determined by using MTT (for PBMCs, 5×10^5^ cells were added each well and the plates were incubated for 7 days). Afterward, the 50% cytotoxicity concentration (CC_50_) was calculated. AZT and NVP were used as positive controls.

### Antiviral activity in C8166

C8166 cells were infected with different HIV-1 laboratory strains and RT inhibitors resistant strains at different serial concentration of compounds with a multiplicity of infection (MOI) of 0.03 as described previously [12]. After 2 hour infection time period at 37°C in a 5% CO_2_ atmosphere, infected cells were washed three times to remove free viruses and resuspended by RPMI-1640 (with 10% FBS). Next, 100 µl of the infected cells (4×10^4^) were then seeded into a 96-well plate, in each well with gradient concentrations of DB-02. AZT and NVP were used as positive controls. On day 3, the p24 levels were measured by in house ELISA [13] and 50% effective concentration (EC_50_) was calculated. 

### Antiviral activity in PBMC

PHA-stimulated PBMCs were incubated with different clinical strains in RPMI-1640 (with 10% FBS, 50 U/ml IL-2 and 2 μg/ml polybrene) at low MOI for 4 hours. Infected PBMCs were then washed three times with PBS, after which 100 μl 5×10^5^ infected cells were seeded in each well of a 96-well plate with gradient concentrations of DB-02. On day 7, p24 antigen levels were measured by ELISA and EC_50_ was calculated.

### Reverse transcriptase activity assay

Reverse transcriptase assay was performed using the Reverse Transcriptase Assay Kit (Roche, Germany) according to the manufacturer’s instructions. 

### Site-directed mutagenesis of reverse transcriptase in HIV-1

Site-directed mutant (SDM) viruses were constructed based on pNL4-3 plasmid. Mutagenesis was performed by using the Fast Mutagenesis System Kit (TransGen; Beijing, China) according to the manual. Five single mutations were used: K103N, V106A, V106M, V108I and Y181C. Antiviral activity against the SDM viruses was tested as described above. 

### Selection of drug-resistant variant virus

The resistant viruses to DB-02 were selected by adding the compound progressively in HIV-1_IIIB_ infected C8166 cells, according to previous described methods, though with some modifications [14,15]. In brief, 1×10^6^ HIV-1_IIIB_ infected C8166 cells (MOI=0.05) were cultured in the presence of 2× EC_50_ of the DB-02 (10 nM). Every 4 to 7 days, when syncytia formation was observed to be over 80%, supernatants were collected. This process was repeated until the compound concentration reached to 40 μM. As a control, HIV-1_IIIB_ infected C8166 cells of the same passages in the absence of DB-02 were carried out in parallel with the cultures.

### Phenotypic and genotypic resistance assay

The selected viruses were used to evaluate the phenotypic resistance as described above. Meanwhile, the RNA of the selected virus was extracted from viral stocks and reverse transcribed to cDNA. PCR amplification was performed as previously reported [16]. A full-length 1680bp of the HIV RT encoding sequence was cloned, and the positive clones were sequenced and then aligned with the wild type HIV-1_IIIB_ strain (GenBank accession number: NC001802).

### Combination antiviral activity assay

Anti-HIV activity of the DB-02 with the NNRTI-nevirapine (NVP), NRTI-zidovudine (AZT), INI-raltegravir (RAL) and PI-indinavir (IDV) were evaluated *in vitro* by using HIV-1_IIIB_ infected C8166 cells after 3 days culture as previously reported [17]. The combination index (CI) was calculated according to the Chou-Talalay Method [18] using Calcusyn 2.0 (Biosoft, USA). A drug combination was marked as synergism if CI<0.8, as antagonism if CI>1.2, and as additive effect if CI between 0.8 and 1.2.

### Molecular docking

Molecular docking studies were performed using AutoDock4.2 program [19]. The X-ray crystal structure of HIV-1 RT (PDB code: 1RT2) was downloaded from the Protein Data Bank (PDB) at the Research Collaboration for Structural Bioinformatics (http://www.rcsb.org/pdb/home/home.do). AutoDockTools 1.5.4 (ADT) were used to prepare the molecules and proteins for docking. The bound ligand and all water molecules were removed from the protein. Gasteiger charges were assigned to both protein and ligands. A grid with spacing of 0.375 Å and 60×60×60 points in the x, y, and z axes was built and centered on the center of mass of the bound ligand in the crystal structure. Energy grid maps for all possible ligand atom types were calculated before performing docking. 

While keeping the protein structure rigid, we selected Lamarckian genetic algorithm (LGA) to search the conformational and orientational space of the ligands. For each small molecule, 100 separate docking calculations were performed with the following settings of parameters: population size of individuals at 350, a maximum number of 25 million energy evaluations, a maximum number of generations of at 27,000, a mutation rate of 0.02, a crossover rate of 0.8, and an elitism value of 1. All other docking parameters were set to default. After docking, cluster analysis was performed on the results with the root-mean-square (RMS) deviations less than 1.0 Å. The best-docked conformation was then selected as the lowest energy pose in the most populated cluster.

## Results

### Antiviral activity of DB-02

To evaluate the antiviral activity of DB-02, the laboratory adapted strains (IIIB, MN) and clinical strains (KM018, TC-2, WAN) were used to infect C8166 cells or PHA-stimulated PBMCs by comparing two current marketed RT inhibitors, NVP (NNRTI) and AZT (NRTI). DB-02 displayed potencies for inhibition of wild-type HIV-1_IIIB_ and HIV-1_MN_ (Table 1), and its EC_50_ values were in the nanomolar range (2.40 to 5.62 nM). In comparison, NVP was less potent (about 3 to 14-fold) than DB-02 in both HIV-1_IIIB_ and HIV-1_MN_ while AZT has a similar EC_50_ value in HIV-1_MN_ (3.35 nM) but with a higher EC_50_ value in HIV-1_IIIB_ (21.8 nM).

**Table 1:** Anti-HIV-1 activity of DB-02 against laboratory adaptive strains, clinical isolated strains, and RT resistant strains^***a***^.

**Strains**	**Subtypes**	**Tropisms**	**Mean EC_50_(nM)^*b*^**				
			**DB-02**		**NVP**		**AZT**
HIV-1_IIIB_	B	X4	2.40±0.27		33.4±18.7		21.8±4.6
HIV-1_MN_	B	X4	5.62±1.35		18.0±5.2		3.35±0.29
HIV-1_KM018_	CRF07_BC	R5	41.8±12.7		135±36		141±56
HIV-1_TC-2_	CRF01_AE	X4	8.74±3.58		96.2±15.8		18.8±5.9
HIV-1_WAN_	CRF07_BC/CRF01_AE	X4	25.0±2.5		100±17		16.5±5.5
HIV-1_A17_ **^*c*^**	B	X4	38,900±18,200		198,000±11,100		16.1±5.7
HIV-1_74V_ **^*d*^**	B	X4	3.27±0.49		39.0±6.3		4.85±0.29

*a* Abbreviations: NVP, nevirapine; AZT, azidothymidine (zidovudine).

*b* Mean activity of EC_50_ was exhibited by mean ± standard deviation, n≥3.

*c* Mutation sites of HIV-1_A17_ are K103N and Y181C in RT encoding region in *pol* gene.

*d* Mutation site of HIV-1_74V_ is L74V in RT encoding region in *pol* gene.

HIV-1 subtype B is widespread in North America and Europe, while some circulating recombinant forms, such as subtype CRF01_AE and CRF07_BC, are more prevalent in China [20,21]. DB-02 also displayed good activity against different subtype and tropism clinical isolates. In particular, the EC_50_ values of DB-02 against HIV-1_KM018_, HIV-1_TC-2_ and HIV-1_WAN_, the major isolates from high AIDS prevalence region in China, were 41.8, 8.74 and 25.0 nM, respectively. By comparison, NVP and AZT were less competent than DB-02, with EC_50_ values were 96.2~135 nM and 16.5~141 nM, respectively.

DB-02 and NVP were both resistant to HIV-1_A17_. In a comparative study with K103N/Y181C double mutation virus, the EC_50_ value of DB-02 was 38.9 and the value for NVP was 198 μM. DB-02 was more sensitive to HIV-1_74V_, a ddI/ddC resistant virus. Our preliminary results also indicated both DB-02 and NVP did not inhibit HIV-2_ROD_ *in vitro* (data not shown).

### Cytotoxicity and therapeutic index of DB-02

**Figure 2:**
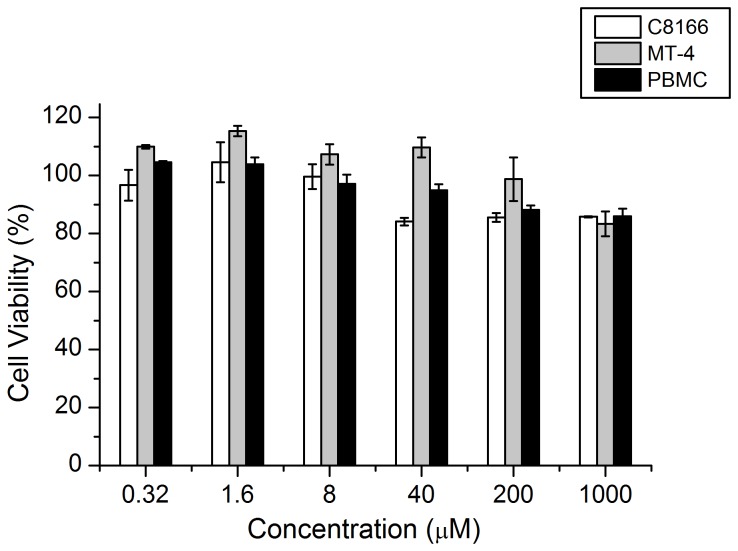
Cytotoxicity of DB-02 in C8166, MT-4 and PBMC using MTT methods. DB-02 showed low cytotoxicity to all the three types of aforementioned cells above. CC_50_ of these cells were all >1000μM, respectively.

MTT methods were used to investigate the toxicity of DB-02 with different cell lines and primary cells. The CC_50_s for DB-02 on C8166, MT-4 and PBMCs cells were all greater than 1000 μM (Figure 2), indicating a cell-based therapeutic index (TI, the ratio of CC50/EC_50_) range of 24,000 to 420,000. These values were rather higher than the control compound NVP. The CC_50_ values of NVP in C8166, MT-4 and PBMCs were 363, 445 and 757μM, respectively, and the TI values were in the range of 5,600~25,000. Similarly, the CC_50_ of AZT varied from 20.1 to 3146 μM, and the TI value was between 650~144,559. Additionally, DB-02 also showed low toxicity in chronic HIV infected H9 cells (CC_50_>1000 μM).

### In vitro reverse transcriptase assay

**Figure 3:**
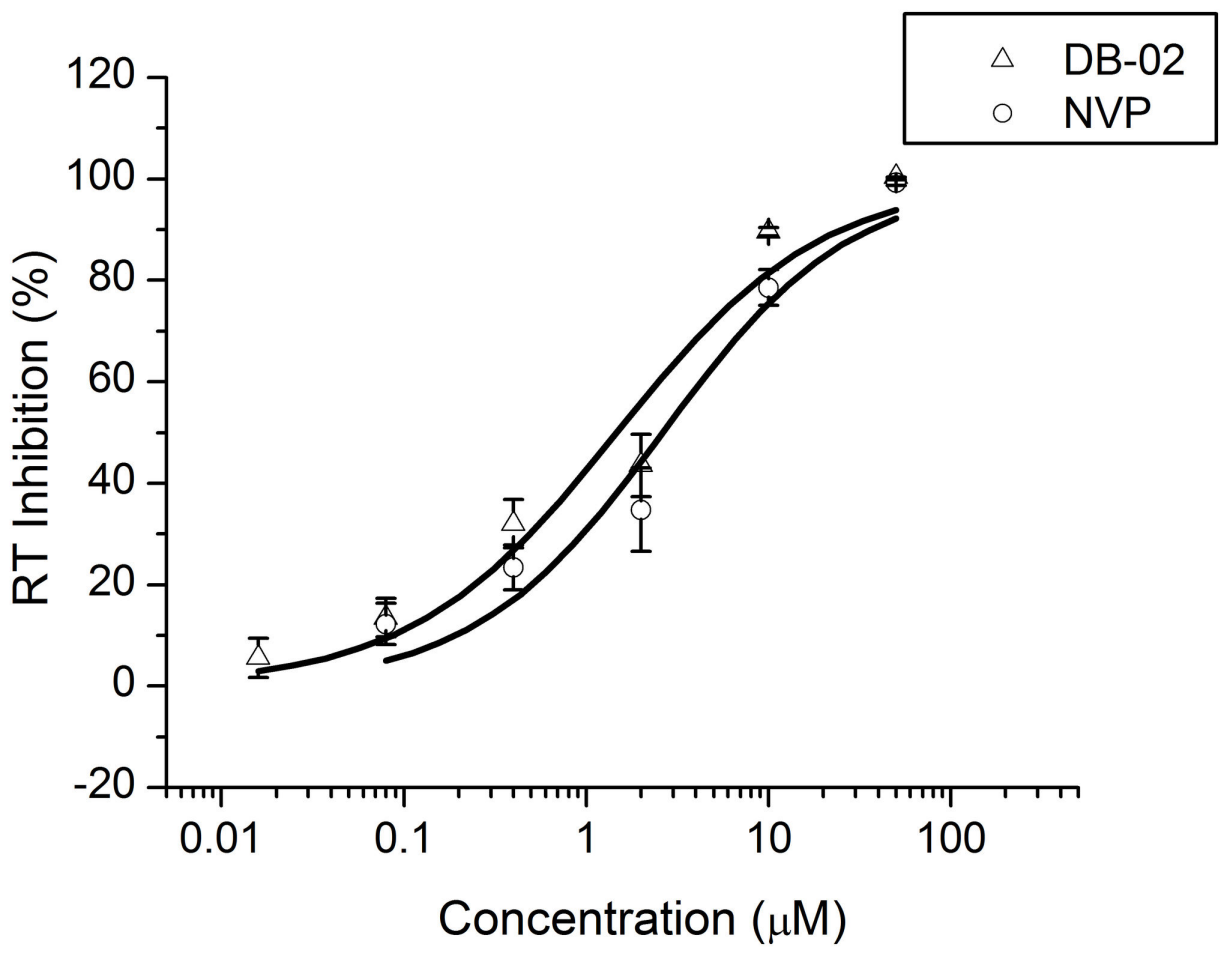
RT activity of DB-2 *in* *vitro*. The RT activity was measured by ELISA using DIG-labeled dNTPs, which were incorporated into the newly synthesized cDNA. The figure represents three independent experiments.

DB-02 has also been tested *in vitro* inhibitory activity against HIV-1 using the DIG labeling ELISA Kit (Roche, Germany). This compound has a similar dose-dependent pattern in inhibiting HIV-1 RT activity with NVP (Figure 3), and the EC_50_s of DB-02 (0.44 ± 0.18 μM) was lower than that of NVP (3.53 ± 1.78 μM).

### Antiviral activity of DB-02 against reverse transcriptase SDM viruses

In this study, five previously clinic reported major amino acids SDM viruses, K103N, V106A, V106M, V108I and Y181C were screened. The assay was measured by p24 ELISA (results summarized in Table 2). Against WT HIV-1_NL4-3_, DB-02 had an EC_50_ of 7.74 nM as compared with 14.35 nM, 0.36 nM and 0.86 nM determined with NVP, EFV and ETR, respectively. The EC_50_s recorded from DB-02 with the five RT mutation viruses ranged from 12.76 nM to 734 nM indicating that one of the mutants, V108I, was effectively inhibited by the compound since the EC_50_ value did not exceed that of the WT virus by more than tenfold. However, other mutants led to a moderate loss of sensitivity for DB-02 within 100-fold shift, including K103N, V106A, V106M and Y181C. Of the single-amino-acid substitutions, the most resistant to DB-02 was V106A. The EC_50_s of NVP were increased more than 10-fold for all the mutants, and four of the five mutants showed over a 100-fold shift; whereas the EC_50_s of EFV were all within 100-fold of the WT value, similar as DB-02. ETR was resistant to Y181C substitution with 12.8-fold change against the WT virus.

**Table 2:** Anti-HIV-1 activity of DB-02 against site-directed mutation viruses^***a***^.

**HIV-1 RT mutants**		**Compounds**										
		**DB-02**			**NVP**			**EFV**			**ETR**	
		**EC_50_(nM)^*b*^**	**FC^*c*^**		**EC_50_ (nM)**	**FC**		**EC_50_ (nM)**	**FC**		**EC_50_ (nM)**	**FC**
WT(HIV-1_NL4-3_)		7.74±0.83	NA**^*d*^**		14.35±4.15	NA		0.36±0.03	NA		0.86±0.20	NA
K103N		709±45	91		1,890±860	135		16.9±7.05	47		1.06±0.09	1.2
V106A		734±117	95		23,500±4,000	1,680		1.66±0.55	4.6		1.79±1.39	2.1
V106M		172±7	22		3,140±40	219		19.8±1.3	55		2.17±0.47	2.5
V108I		12.76±0.42	1.6		440±50	31		0.98±0.14	2.7		1.91±1.05	2.2
Y181C		241±35	31		3,320±2,710	237		0.82±0.65	2.3		11.0±8.69	12.8

*a* Abbreviations: NVP, nevirapine; EFV, efavirenz; ETR, etravirine.

*b* EC_50_s are exhibited by means ± standard deviations, n≥3.

*c* FC, fold change. Calculated as the mean EC_50_ of mutants divided by wild type HIV-1_NL4-3_.

*d* NA, not applicable.

### Phenotypic resistance assay of DB-02 from in vitro selection

To investigate *in vitro* drug-resistance of DB-02, HIV-1_IIIB_ infected C8166 cells were cultured with DB-02 by concentration doubled every 5-7 days until it reached 40 μM. The antiviral activity against the induced-resistance virus was determined by the phenotypic resistance assay. The EC_50_ of DB-02 for selected virus was 274±70.7 μM (32,888 fold change in comparison with wild-type HIV-1_IIIB_), which was consistent with the highest concentration used in the selection culture (Table 3). However, NVP did not show any inhibitory activity at its nontoxic concentration (CC_90_=70.0±23.4 μM, lower than EC_50_>400 μM). EFV and ETR also showed antiviral activity with slight resistance in the nanomolar range, with EC_50_s of 20.1±2.7 nM and 18.8±1.2 nM, respectively.

**Table 3:** Antiviral activity against DB-02 induced resistant virus^***a***^.

**Strain**	**Mean EC_50_ (nM)^*b*^**						
	**DB-02**		**NVP**		**EFV**		**ETR**
HIV-1_DB-R_	274,000±70,700		>400,000		20.1±2.7		18.8±1.2

*a* HIV-1_IIIB_ was used in selection culture by treatment of DB-02.

*b* EC_50_s are exhibited by means ± standard deviations, n≥3.

### Genotypic resistance assay of DB-02 from in vitro selection

To determine which amino acid was modified *in vitro* in presence of DB-02, the full RT sequence (1680 bp) was analyzed from the DB-02-selected virus genome, as well as the wild-type control. Thirty-three positive clones were picked and 30 mutation sites in total were observed based on clones sequencing (Table 4). As demonstrated in the SDM assay, V106A, the highest resistant mutant to DB-02, displayed a 100% mutation frequency among all the mutation sites. Y181C, a moderate resistance site, exhibited 91% mutation frequency. R461K and Y483H showed moderate mutation frequency of 41% and 24%, respectively in the RNase H domain. S162N/G, V179D and L214F had a lower mutation frequency of 9%. The remaining 23 mutation sites showed very low frequencies, lower than 6% (Table 4). 

**Table 4:** Genotypic patterns of HIV-1_IIIB_ selected by DB-02.

**Mutation sites^*a*^**	**No. of clones**	**Mutation frequency (%)^*b*^**
V106A	all	100
S162N/G	8,24,33	9
V179D	7,20,30	9
Y181C	1,2,3,4,5,6,8,9,10,11,12,13,14,15,16,17,18,19,20,21,22,23,25,26,27,28,29,31,32,33	91
L214F	12,21,26	9
R461K	1,3,4,6,11,12,14,16,19,20,26,31,32,33	41
Y483H	7,14,15,21,22,23,29,31	24

*a* Mutation sites of amino acids are aligned with HIV-1_IIIB_.

*b* Other sites with mutation frequency lower than 6% include the following: V21I, K43R, K46R, P133S, T165A, D177G, D185G, D218G, V254A, H221Y, L228F, G316R, Y339H, L349M, V381I, T409A, V423M, S447N, A485T, S489P, I495T, A508T, Q512K, L517S and K528E.

### Antiviral activity of DB-02 in combination with other drugs

In order to verify the efficiency of DB-02 in combination with other drugs, DB-02 was tested in combination studies along with four FDA-approved drugs. These drugs presenting four types of ARVs drugs: NVP (NNRTI), AZT (NRTI), RAL (INI), and IDV (PI). The combination index (CI) was calculated using Calcusyn 2.0 software. As expected, DB-02 showed no signs of antagonism with the four drugs in this test. AZT was synergistic with DB-02 while DB-02 was additive with NVP, RAL and IDV (Table 5).

**Table 5:** Antiviral activity for DB-02 in combination with four FDA-approved antiviral compounds against HIV-1_IIIB_.

**Drug type^*a*^**	**Drug^*b*^**	**CI^*c*^**	**Interaction**
NNRTI	NVP	0.95±0.03	Additive
NRTI	AZT	0.61±0.16	Synergy
INI	RAL	1.03±0.15	Additive
PI	IDV	1.15±0.06	Additive

*a* NNRTI, non-nucleoside reverse transcriptase inhibitor; NRTI, nucleoside reverse transcriptase; INI, integrase inhibitor; PI, protease inhibitor.

*b* NVP, nevirapine; AZT, azidothymidine; RAL, raltegravir; IDV, indinavir.

*c* CI, combination index, is showed by mean ± standard deviation, n≥3.

### Molecular docking

**Figure 4:**
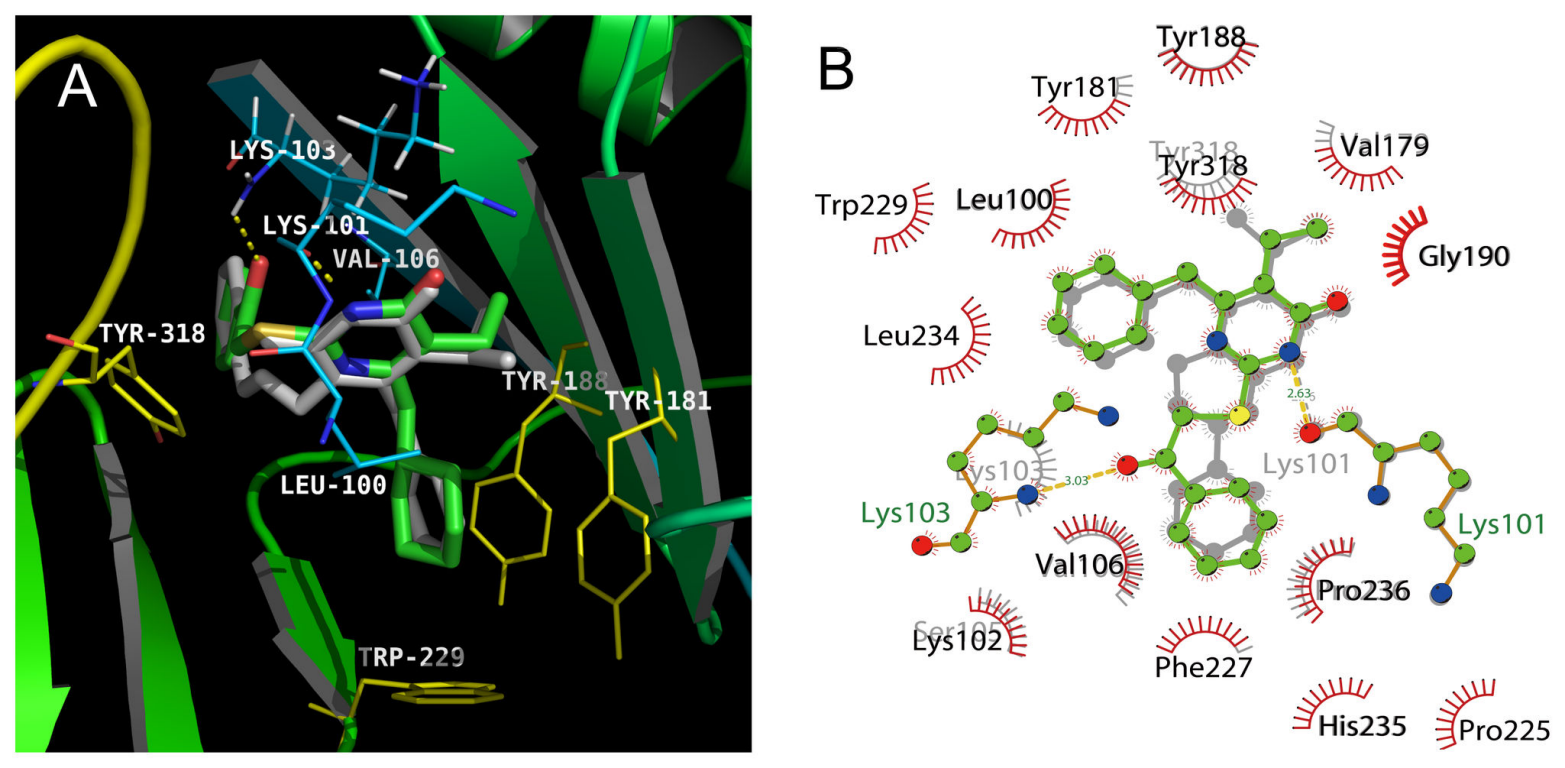
Molecular docking of DB-02. (**A**) Superimposition of the docked conformations of DB-02 (green-colored carbon atoms) and TNK-651 (grey). The docking results are showed by PyMOL. The backbone is represented by ribbons, and amino acid residues important for binding interactions are labeled. Dotted lines show the interactions between HIV-1 RT and DB-02. (**B**) The two-dimension representations of the interactions between the NNIBP and DB-02 (green) or TNK-651 (grey) are presented after the docking.

The molecular modeling study was performed using Autodock Vina. Due to the high structural similarity between HIV-1 RT/6-benzyl-1-(benzyloxymethyl) -5-isopropylpyrimidine-2,4(1H,3H)-dione (TNK-651) and DB-02, three-dimensional coordinates of the TNK-651 complex (Brookhaven Protein Data Bank entry 1RT2) were used as the input structure for docking calculations. The theoretical binding modes of DB-02 to the NNRTI binding pocket (NNIBP) compared with TNK-651 are shown in Figure 4A and 4B. The docking simulation showed the binding mode of the DB-02 into the NNIBP: (1) the pyrimidine ring of compound DB-02 and TNK-651 overlapped very well. Both the pyrimidine NH moiety at position 3 of DB-02 and TNK-651 were engaged in a hydrogen bond with the C=O moiety of Lys101; (2) the 2-phenylcarbonylmethylthio substituent of DB-02 were well accommodated in the large pocket mainly defined by Val106, Pro236, Phe227, Leu234, His 235 and Tyr318, in spite of there are different orientation of the C-2 side chain of DB-02 and the *N*-*1* side chain of TNK-651, the terminal phenyl rings are located in the same area of the NNIBP. Good profitable hydrophobic interactions were found between the hydrophobic side chains of Val106, Leu234, His 235, Tyr318 and the phenyl ring of the C-2 or *N-1* side chain; compared with TNK-651, another hydrogen bond was formed between the C=O group of the C-2 side chain of DB-02 and NH group of Lys103 main chain (3.03 Å) which is good for improving the activity of compounds; and (3) the cyclohexyl substituent at *C*-*6* of DB-02 and the C-6-benzyl of TNK-651 are positioned in the same subpocket of the NNIBP, which is formed by Leu100, Tyr181, Tyr188, Val106, and Trp229. In particular, the C-6-phenyl ring of TNK-651 interacts favorably with the Tyr181 and Tyr188 side chains, and results a positive π-stacking interaction. While the C-6-cyclohexyl ring of DB-02 adopted the lowest-energy ‘chair’ conformation fitting deeper into the hydrophobic region of the NNRTI pocket and made numerous hydrophobic interactions and van der Waals contacts to residues of Tyr181, Tyr188, Val106 and Trp229, which play an important role in the interactions between the RT and the inhibitors.

## Discussion

In this study, we evaluated DB-02 antiviral activities against different HIV-1 viruses, including laboratory-adaptive strains, RT resistant strains, and clinical isolated predominant strains from HIV-1 virus subtypes CRF_01AE and CRF07_BC (prevalent HIV-1 viruses in China). DB-02 was found to be highly sensitive against laboratory-adaptive strains and clinical isolated strains, with all EC_50_s lower than 50 nM. This finding indicates that DB-02 has more effective anti-HIV-1 activities than NVP, and a similar activity to EFV and ETR. Antiretroviral therapy for the treatment of HIV infection requires high levels of adherence to both maintain plasma HIV RNA at undetectable levels and prevent the emergence of drug resistance [22]. One adverse effect of NVP was its decreased adherence to AIDS patients which has become one of the major causes of NNRTI treatment failure. Toxicity is another adverse effect of NNRTI treatment that should be put into consideration. DB-02, however, exhibited very low cytotoxicity to T-cell lines and primary PBMCs, better than NVP and the major groups of DABOs compounds [23-25].

We also analyzed the resistance spectrum by the known resistant strains and DB-02 selected viruses. DB-02 and NVP were highly sensitive to HIV-1_74V_, an NRTI resistant strain. Although both DB-02 and NVP showed high degree of resistance to HIV-1_A17_, a double mutation strain with K103N and Y181C, DB-02 showed effective antiviral activity at very low toxic concentration. 

To date, there are five NNRTIs approved by FDA with chemical heterogeneity. They all bind at the same hydrophobic pocket site of the RT [26]. We tested DB-02 antiviral activities against five single site-directed mutants: K103N, V106A, V106M, V108I and Y181C, the sites participate in the formation of the hydrophobic pocket. Compared to other NNRTIs, DB-02 was sensitive to V108I, and had a moderate resistance to K103N, V106A, V106M and Y181C. DB-02 displayed a better anti-resistance activity than NVP but less than EFV and ETR. NVP had high or moderate resistance level, indicating its antiviral activity against single-amino-acid substitutions was worse than DB-02, EFV and ETR. These results suggest that DB-02 has higher sensitivity against RT mutants than NVP does. 

Selection of drug induced mutants *in vitro* indicated that the DB-02 induced viruses were resistant to NVP, but still sensitive to EFV and ETR (Table 3). This indicates that EFV and ETR may still be effective after DB-02 treatment, due to their torsional flexibility in the hydrophobic pocket even with some mutations [27]. This selected pattern *in vitro* could help us understand how the compound interacts with the enzyme by analyzing its genetic information. Our results (Table 4) suggest that the DB-02 induced dominant mutations are V106A (100%), Y181C (91%), R461K (41%), S162N/G (9%), V179D (9%) and L214F (9%). Previous studies have indicated that V106A and V179D were low in prevalence in NNRTI-resistant samples, with frequencies of 2.37% and 3.25%, respectively, compared with the high prevalence of K103N (56.95%) and Y181C (24.95%), the most widely recognized NNRTI-resistance mutations [5]. Although K103N was the most common NNRTI mutation in subjects taking combination ART with NVP and AZT [28], it was not observed during our 22 passages of culture in the presence of DB-02. Y181C is cross-resistant to all the NNRTIs approved for clinical use at different resistance levels [29] and similar observation had been obtained with DB-02. This suggests that Tyr181 is located in a key position of the “pocket” and is quite difficult to circumvent. On the other hand, R461K has only been rarely reported until recently, and was considered a result of polymorphisms of RT not related to any NNRTI resistance, since several HIV-1 (subtype B) prototype strains contain Lys461 in the RNase H domain [30]. In addition, L214F, Y483H and Q512K occur naturally as a polymorphism and do not cause NNRTI resistance [15]. Other mutations with frequency of <6%, might be produced by error-prone reverse transcriptase during viral genome synthesis, or possibly from misincorporations by cellular RNA polymerase II during viral RNA synthesis [31,32].

Multiple drug combinations result in durable control of HIV-1 replication when compared with single use. There was no evidence of antagonistic interactions between DB-02 and other compounds tested (Table 5). Among all the drugs employed in the assay, NVP, RAL and IDV showed additive effects with DB-02 and AZT showed synergism to DB-02. 

We further explored the binding sites of RT based on *in silico* analysis. Although they are structurally distinct, all the NNRTIs bind to a common allosteric site of HIV RT about 10Å from the catalytic site, and interfere with RT by altering either the conformation or the mobility of RT. There are several interactions occurring within the allosteric pocket, the π-π interaction with Tyr181 residue appears to be essential for the inhibitory effect on HIV-1. When Tyr181 is mutated to cysteine residues, the inhibitory activity decreased, due to the loss of the important aromatic ring interactions in the core of the NNRTI-binding pocket. However, the nonaromatic cyclohexyl group of DB-02 is not restricted by the loss of the π-π stacking. In the genotypic resistance assay, Val106 was found to be important (100% mutation frequency) since there was a van der Waals interaction between DB-02 and Val106 (Figure 4B). DB-02 has two hydrogen-bond interactions with Lys101 and Lys103. The hydrogen bond between the NH and C=O groups located along the protein backbone and not with the amino acid side chain. When the Lys103 was mutated to Asn, the side chain becomes oriented away from but the main chain shows little change in orientation. This might partially explain why DB-02 only lost 91-fold activity against K103N mutant. Since hydrogen bond interactions are more stable than van der Waals interactions, DB-02 can still retain weak activity against K103N+Y181C. Interestingly, we did not observe the Lys101 or Lys103 mutation in DB-02 induced mutation assay. More investigations are needed to explain this observation.

Since DABO family exhibits robust antiviral activity for their high specificity and low toxicity [6,33], we compared 456 *S*-DABO analogs and summarized 20 of them with special high antiviral activity (TI >20,000) in Table 6 [7-9,25,34-41]. Among these analogs, compound **19**, reported by Mugnaini et al., could reach an excellent activity at picomolar (EC_50,_W_T_ =25 pM) level, indicating that *S*-DABO family is might be a good candidate of NNRTIs [25]. The latest research on *S*-DABOs reported by Radi et al., released a very potent agent, compound **2c *(R,R,R***), showing high activity against WT virus (TI=209,000) and moderate activity against K103N or Y181C (FC of 514 or 185, respectively) [38]. However, most of their inhibitory activity would be largely diminished by K103N or Y181C mutation. Notably, DB-02 showed an improved sensitivity against K103N or Y181C with FC lower than 100, the best competent one in the table. Such improvements encourage us further to explore *S*-DABO analogs and discover more potent compounds against such mutations. 

**Table 6:** Recent *S*-DABOs with high inhibitory activity in comparison of DB-02^***a***^.

**Compound^*b*^**	**TI^*c*^**		**FC^*d*^**			**Reference**
			**K103N**	**Y181C**		
DB-02	>420,000		91	31		
**3s**	29,000		ND**^*e*^**	ND		[36]
**3v**	>20,000		ND	ND		[36]
**3w**	>33,000		ND	ND		[36]
**[+]-3w**	>100,000		ND	ND		[36]
**20**	>407,000		>6,214	ND		[37]
**2**	>26,000		3,177	377		[25]
**9**	>56,000		32,850	18,800		[25]
**10**	>54,000		4,100	1,200		[25]
**19**	>497,000		22,000	12,000		[25]
**24**	>301,000		9,615	13,900		[25]
**47**	>59,000		1,300	650		[25]
**49**	28,000		5,100	3,500		[25]
**58**	28,000		836	590		[25]
**5p**	>32,000		ND	ND		[7]
**6l**	>35,000		35,000	575		[9]
**7e**	>30,000		4,360	140		[9]
**8g**	>30,000		2,230	120		[9]
***rac*-2**	62,000		866	266		[38]
**2c(R,R,R)**	209,000		514	185		[38]
**1**	>414,000		18,333	10,000		[41]

*a* Totally, 456 *S*-DABO compounds were investigated, only those with high activity(TI>20,000) were listed in the table.

*b* Compound names are cited directly from the references. If two compounds are in the same name, please check related reference for details.

*c* TI, therapeutic index, the ratio of CC50/EC_50_.

*d* FC, fold change, the ratio of EC_50(Mut_)/EC_50(WT)_.

*e* ND, no data in the reference.

In conclusion, the present study highlights a new highly functional *S*-DACO derivatives, DB-02 with high potency (EC_50_s at nanomolar level) and low cytotoxicity (CC_50_s > 1mM) against HIV-1 reverse transcriptase. *In vitro* selection, site-directed mutagenesis and molecular docking demonstrate that Val106 is the most important location for the compound to be effective. DB-02 was also shown to be non-antagonistic with other anti-HIV drugs. The results of this study further indicated that DB-02 is a potential NNRTI that has low cytotoxicity and improved activity towards RT mutants other than *S*-DABO analogs. Likewise, this research could also provide clues in further optimizing *S*-DACOs in order to obtain a higher genetic barrier and lower cytotoxicity. The knowledge gained from this study may then be helpful in development of future drug designs, allowing the synthesis of higher potent compounds targeted to block HIV-1 reverse transcriptase.
